# Dissociable Temporo-Parietal Memory Networks Revealed by Functional Connectivity during Episodic Retrieval

**DOI:** 10.1371/journal.pone.0071210

**Published:** 2013-08-29

**Authors:** Satoshi Hirose, Hiroko M. Kimura, Koji Jimura, Akira Kunimatsu, Osamu Abe, Kuni Ohtomo, Yasushi Miyashita, Seiki Konishi

**Affiliations:** 1 Department of Physiology, The University of Tokyo School of Medicine, Tokyo, Japan; 2 Department of Radiology, The University of Tokyo School of Medicine, Tokyo, Japan; 3 Department of Physiology, Juntendo University School of Medicine, Tokyo, Japan; 4 Precision and Intelligence Laboratory, Tokyo Institute of Technology, Yokohama, Japan; Nathan Kline Institute and New York University School of Medicine, United States of America

## Abstract

Episodic memory retrieval most often recruits multiple separate processes that are thought to involve different temporal regions. Previous studies suggest dissociable regions in the left lateral parietal cortex that are associated with the retrieval processes. Moreover, studies using resting-state functional connectivity (RSFC) have provided evidence for the temporo-parietal memory networks that may support the retrieval processes. In this functional MRI study, we tested functional significance of the memory networks by examining functional connectivity of brain activity during episodic retrieval in the temporal and parietal regions of the memory networks. Recency judgments, judgments of the temporal order of past events, can be achieved by at least two retrieval processes, relational and item-based. Neuroimaging results revealed several temporal and parietal activations associated with relational/item-based recency judgments. Significant RSFC was observed between one parahippocampal region and one left lateral parietal region associated with relational recency judgments, and between four lateral temporal regions and another left lateral parietal region associated with item-based recency judgments. Functional connectivity during task was found to be significant between the parahippocampal region and the parietal region in the RSFC network associated with relational recency judgments. However, out of the four tempo-parietal RSFC networks associated with item-based recency judgments, only one of them (between the left posterior lateral temporal region and the left lateral parietal region) showed significant functional connectivity during task. These results highlight the contrasting roles of the parahippocampal and the lateral temporal regions in recency judgments, and suggest that only a part of the tempo-parietal RSFC networks are recruited to support particular retrieval processes.

## Introduction

Retrieval of past episodes recruits multiple processes, such as recollection and familiarity during item recognition, and these processes are thought to involve different regions in the temporal lobe [1]–[9]. Previous studies suggest that the left lateral parietal cortex also contains dissociable regions that are associated with the multiple retrieval processes [5], [10]–[17]. Moreover, studies of resting-state functional connectivity (RSFC) have revealed significant connectivity between the temporal and parietal regions that may support the retrieval processes [18]–[21]. The temporo-parietal networks can also be interpreted as a possible candidate for the neural substrates of the bottom-up and top-down attention that facilitate memory retrieval through the interaction with memory-related activity in the temporal lobe [22].

Analyses of RSFC have been proved to be a powerful tool for revealing large-scale networks of functional interactions among brain regions [23]–[35]. It is well known that regions that are activated during a task tend to be connected with each other in the RSFC network [36]–[44]. However, the task-related brain activity in the regions of the RSFC network during particular psychological processes may not show functional connectivity [45], which is considered more directly linked to neural mechanisms supporting the psychological processes. It is to be elucidated whether the task-related brain activity in the temporo-parietal memory RSFC network shows functional connectivity to support the memory retrieval processes required in the task.

In the present functional MRI study, we examined functional connectivity based on inter-subject correlation of the brain activity during task between the regions of interest (ROIs) in the RSFC networks. A recency judgment paradigm was employed where two studied items were judged as to which was presented more recently [20], [46]–[63]. Recency judgments can be achieved by multiple strategies [64]–[66]. In particular, recency judgments can be based on retrieval of the relations among studied items and the studied items themselves (relational and item-based recency judgments, respectively) [20], [56], [62], [67]. Relational recency judgments involve retrieval of detailed temporal and relational contexts that can be used to bridge between the paired items. Item-based recency judgments are made based on the difference in the strength of familiarity of the paired words or the distinctiveness of the item located in end positions. However, it is to be noted that item-based recency judgments may not share their neural mechanisms with familiarity processes of item recognition. In the present study, we first identified the RSFC-based temporo-parietal networks between temporal and parietal regions associated with relational and item-based recency judgments. We then calculated task-related functional connectivity of the temporo-parietal RSFC networks by examining the inter-regional correlation of brain activity during relational and item-based recency judgments.

## Materials and Methods

### Subjects and fMRI procedures

Written informed consent was obtained from 72 healthy right-handed subjects (37 males; 35 females, age: 20–24 years). Whole experimental procedures were approved by the institutional review board of the University of Tokyo School of Medicine. Scanning was conducted using a 1.5 T fMRI system. Scout images were first collected to align the field of view centered on the subject's brain. Then T2-weighted spin-echo images were obtained for anatomical reference (TR  = 5.5 sec, TE  = 30 msec, 75 slices, slice thickness  = 2 mm, in-plane resolution  = 2×2 mm). For functional imaging, gradient echo echo-planar sequences were used (TR  = 3 sec, TE  = 50 msec, flip angle  = 90 deg, cubic voxel of 4 mm, 22 slices with 2 mm gaps).

### Behavioral procedures

**Figure 1:**
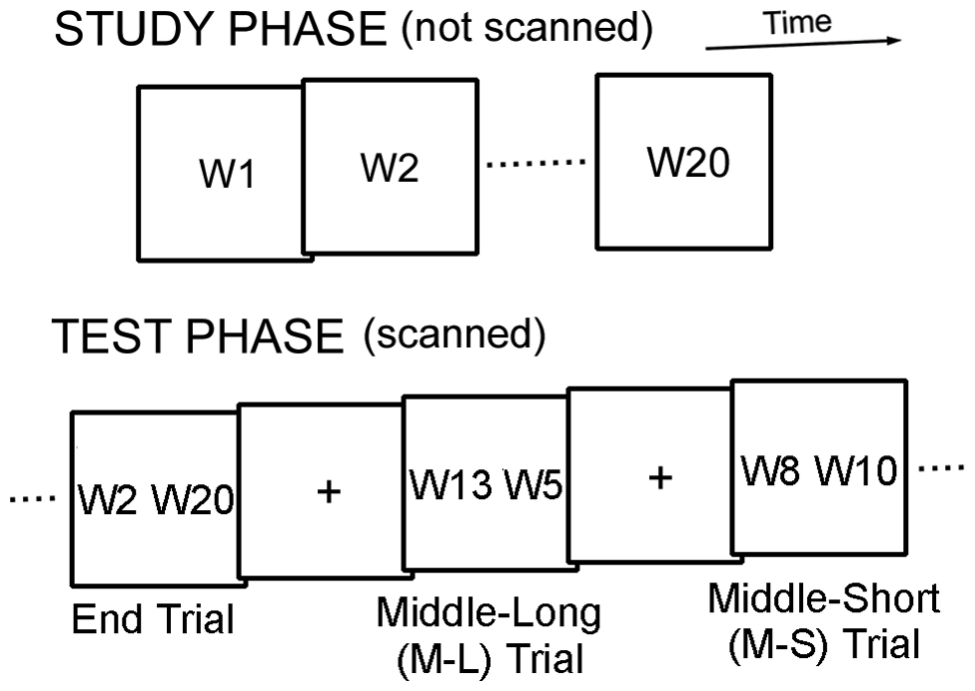
The recency judgment task used in the present study. The task contained three types of trials during the test phase that differed in terms of the presence/absence of presentation of end words and the temporal distance between presented words: End trials, Middle-Long (M-L) trials, and Middle-Short (M-S) trials. W: Word.

Visual stimuli were presented to subjects by projecting them onto a screen. Subjects viewed the screen through prism glasses. A magnet-compatible four-channel button press based on a fiber-optic switch was used to record subjects' performance. The recency judgments task consisted of two main phases, study and test (Fig. 1). During the study phase, twenty words were sequentially presented. Each word was presented for 1 sec, with an inter-stimulus interval (presentation of a white fixation cross) of 1 sec. Subjects were instructed to intentionally encode them for later recency judgments [56], [68]. More specifically, subjects were instructed to make up their own story from the list words, and this instruction is supposed to encourage the subjects to relate sequentially presented words that had otherwise no contexts among them. The words were concrete nouns taken from an object stimulus set [69] and were presented in strings of Japanese characters. To prevent the subjects from rehearsing the words between the study and test phases, the subjects performed a modified Wisconsin card sorting task [70] for approximately 30 seconds as a distracter task [52].

The test phase was administered immediately (∼30 sec) after the distracter task while functional images were acquired. In one recency judgment trial, two words in the studied list were simultaneously presented, one to the right and the other to the left for 3 sec plus 1 sec fixation (Fig. 1). The subjects were instructed to choose which word had been studied more recently. The right or left word was chosen by pressing a right or left button, respectively, using the same right thumb. There were three types of trials of interest that differed in terms of the presence/absence of presentation of one of the two end words and the temporal distance between presented words, that is, End trials, Middle-Long (M-L) trials, and Middle-Short (M-S) trials. The paired words in End trials contained an end word, and were separated by 18 words. The specific pairs in End trials were W1-W19 and W2-W20. The paired words in M-L and M-S trials did not contain an end word, and were separated by 8 and 2 words, respectively. The specific pairs in Middle trials were W3-W11, W4-W12, W5-W13, W6-W14, W7-W15, W8-W16, W9-W17 and W10-W18 for M-L trials, and W3-W5, W4-W6, W7-W9, W8-W10, W11-W13, W12-W14, W15-W17 and W16-W18 for M-S trials. Therefore, retrieval of detailed temporal and relational contexts needed to be recruited to judge the precise temporal order of the pair in M-L and M-S trials, particularly in M-S trials, whereas the difference in the strength of familiarity of the paired words or the distinctiveness of the item located in end positions could be used to judge the temporal order in End trials. Thus, the contrast of Middle (average of M-L and M-S) minus End trials is expected to reveal the brain activity associated with relational recency judgments, whereas the reverse contrast of End minus Middle trials is expected to reveal the brain activity associated with item-based recency judgments [20]. Twelve runs were administered to the subjects, and each one run contained ten recency judgment trials: two End trials, four M-L trials and four M-S trials, plus two fixation trials where a fixation cross was presented throughout the trials. Two separate sets of word lists were used for practice to familiarize the subjects with the entire procedures.

A separate set of 17 subjects (10 males; 7 females, age: 20–24 years) was recruited for an additional behavioral experiment (not scanned) to analyze self-report of retrieval strategies. The task was basically the same, except for that, immediately after recency judgment period in the test phase, there was a self-report period for 4 sec, during which the subjects were asked about the retrieval strategy that they took in the last recency judgments. They were visually presented with three alternatives (“relation”, “item” in Japanese, and “?”) in the PC monitor, and were instructed to choose one of the three: (1) judgment based on relational contexts that they created during encoding, (2) judgment based on item-based information such as relative strength of the paired words and distinctiveness of an end word, and (3) just guess or other retrieval strategies. The word lists employed in this behavioral experiment were the same as those used in the main experiment. After completion of the twelve sets of word lists, they were asked, regarding the alternative 3, whether they used retrieval strategies other than the relational and item-based ones, but none of them reported a new strategy. Two separate practice word lists were used to familiarize the subjects with the entire procedures.

### Data analysis

Data were analyzed using SPM2 (http://www.fil.ion.ucl.ac.uk/spm/). Functional images were realigned, slice timing corrected, normalized to the default template with interpolation to a 2×2×2 mm space, and spatially smoothed (FWHM  = 8 mm). Then event timing was coded into a general linear model (GLM) [71], [72]. The three types of events of interest, correct End, correct M-L, and correct M-S trials, together with other effects of no interest including error trials and run-specific effects, were coded using the canonical hemodynamic response function, time-locked to the onset of these trials. Images of parameter estimates for the signal response magnitude were analyzed in the second-level group analysis using a random effect model. Peak coordinate locations in activation maps were generated using a threshold of P<.05 (FWE corrected for multiple comparisons). Four critical activations reported in Kimura et al. [20] were used for small volume correction: two associated with relational recency judgments (Middle vs. End) in the temporal cortex (−32, −42, −12) and in the parietal cortex (−38, −78, 30), and two associated with item-based recency judgments (End vs. Middle) in the temporal cortex (50, 8, −32) and in the parietal cortex (−56, −60, 42). The temporal and parietal activations in the present study cleared P<.05 corrected for multiple comparison for a small volume based on these previously reported coordinates. All the coordinates are presented using MNI space.

Functional connectivity based on inter-subject correlation of brain activity during task [73]–[75] was also calculated between regions based on the brain activity during Middle minus End trials by correlating individual beta-weights. The magnitude of the brain activity for each subject was averaged across all the voxels in the spherical (r = 8 mm) ROIs that were selected from the temporal and parietal regions activated during Middle minus End or End minus Middle trials. The magnitude of the brain activity for each subject was plotted against a pair of ROIs, and the Pearson's correlation coefficients between the ROIs were calculated. The underlying rationale is that there is meaningful structure in the inter-subject variability which can be explored by assuming that regions belonging to the same network will have comparable variations from subject to subject, and that regions that co-vary across subjects can be considered as part of the same network [73]–[77].

The resting-state fMRI data set of 51 subjects (27 males; 24 females, age: 20–28 years) was collected from our previous study (25 subjects) [20], [78] and the present study (26 subjects). The data analysis procedures for RSFC were essentially the same as those used in previous literatures [25], [37]. Briefly, the acquired images were realigned, slice-timing corrected, and normalized to the standard template image. The images were subject to further preprocessing including temporal band-pass filter (0.009 Hz < f<0.08 Hz), spatially smoothed (FWHM  = 8 mm), regression of six parameters obtained by head motion correction, whole brain signal averaged over the whole brain, ventricular signal averaged from ventricular ROI, and white matter signal averaged from white matter ROI. Functional connectivity analyses were performed on the resultant time series data, on a timepoint by timepoint basis. To estimate the statistical significance of the functional connectivity, the Fischer z transformation was applied to the correlation coefficients [37]. The z-values were calculated between a seed ROI and a target ROI or between a seed ROI and all the voxels in the whole brain. The time series in the seed/target ROI were averaged across all the voxels in the sphere (r = 8 mm) that were selected from the temporal and parietal regions activated during Middle minus End or End minus Middle trials.

## Results

### Behavioral results

**Figure 2:**
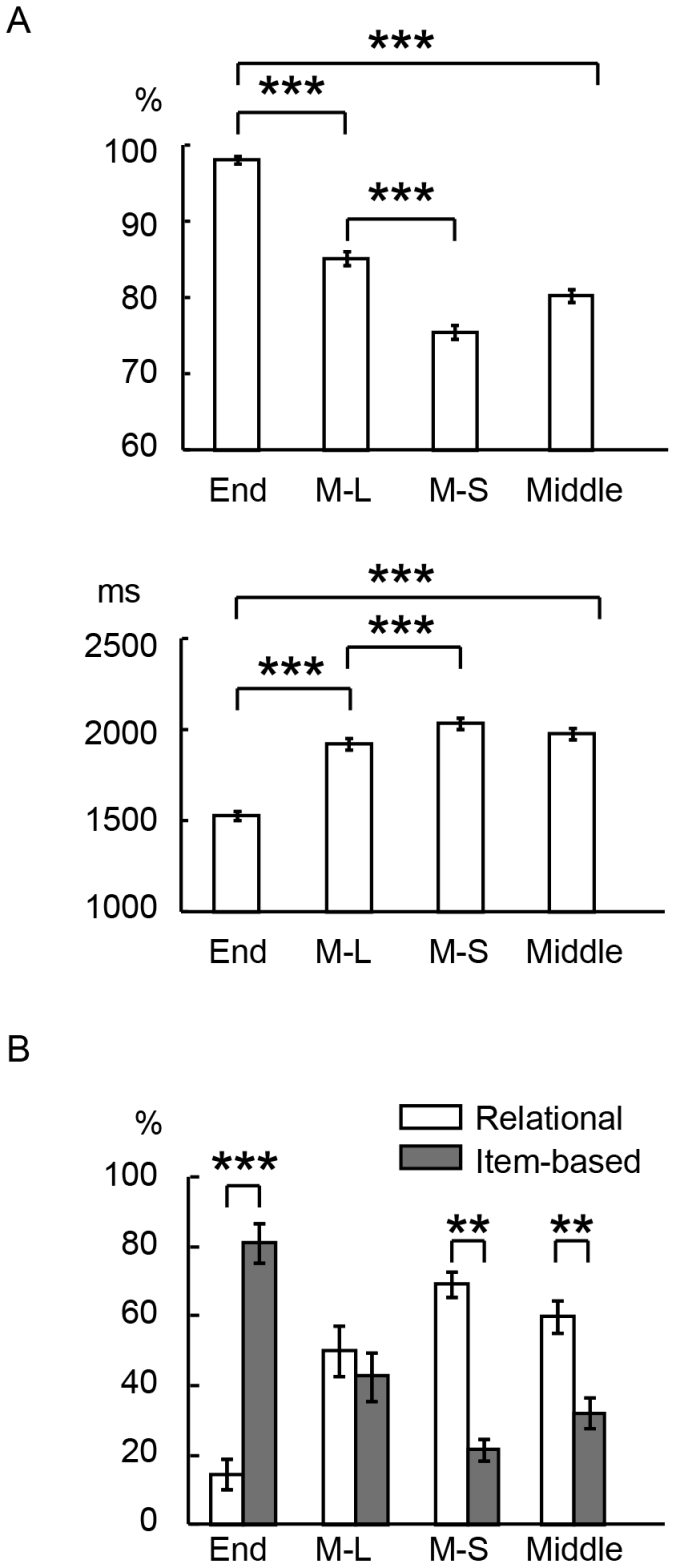
The behavioral results of the recency judgment task. A) Correct performance and reaction times in the three types of the recency judgment trials: End, M-L, M-S and Middle (average of M-L and M-S) trials. ***: P<.001. B) Percentage of trials where relational and item-based strategies were employed. **: P<.01, ***: P<.001.

The correct performance was 98.1±4.1 (mean ± SD), 85.2±8.2 and 75.5±9.1%, in End, M-L and M-S trials, respectively (Fig. 2A top). The difference was significant between End and M-L trials [t (71)  = 15.5, P<.001], between M-L and M-S trials [t (71)  = 11.5, P<.001] and between End and Middle (average of M-L and M-S trials) [t (71)  = 24.3, P<.001]. The reaction time was 1529±216 (mean ± SD), 1922±261 and 2035 ± 265 ms, in End, M-L and M-S trials, respectively (Fig. 2A bottom). The difference was significant between End and M-L trials [t (71)  = 23.8, P<.001], between M-L and M-S trials [t (71)  = 8.7, P<.001] and between End and Middle trials [t (71)  = 27.5, P<.001].

Figure 2B shows the percentage of trials where relational and item-based strategies were employed in End, M-L and M-S trials. In End trials, subjects employed relational and item-based strategies in 14.4% and 81.4%, respectively, and the difference was significant [t (16)  = 6.8, P<.001]. In M-L trials, similarly, the percentages were 50.1% and 42.7%, and the difference was not significant. In M-S trials, the percentages were 69.4% and 21.6%, and the difference was significant [t (16)  = 7.4, P<.001]. In Middle trials, the average of M-L and M-S, the percentages were 59.8% and 32.1%, respectively, and the difference was significant [t (16)  = 3.0, P<.01]. Two-way ANOVA with strategy and trial type as factors revealed significant interaction [F (1, 16)  = 85.9, P<.001]. These behavioral results suggest that the subjects employed relational and item-based strategies dominantly in Middle and End trials, respectively.

### Neuroimaging results

**Figure 3:**
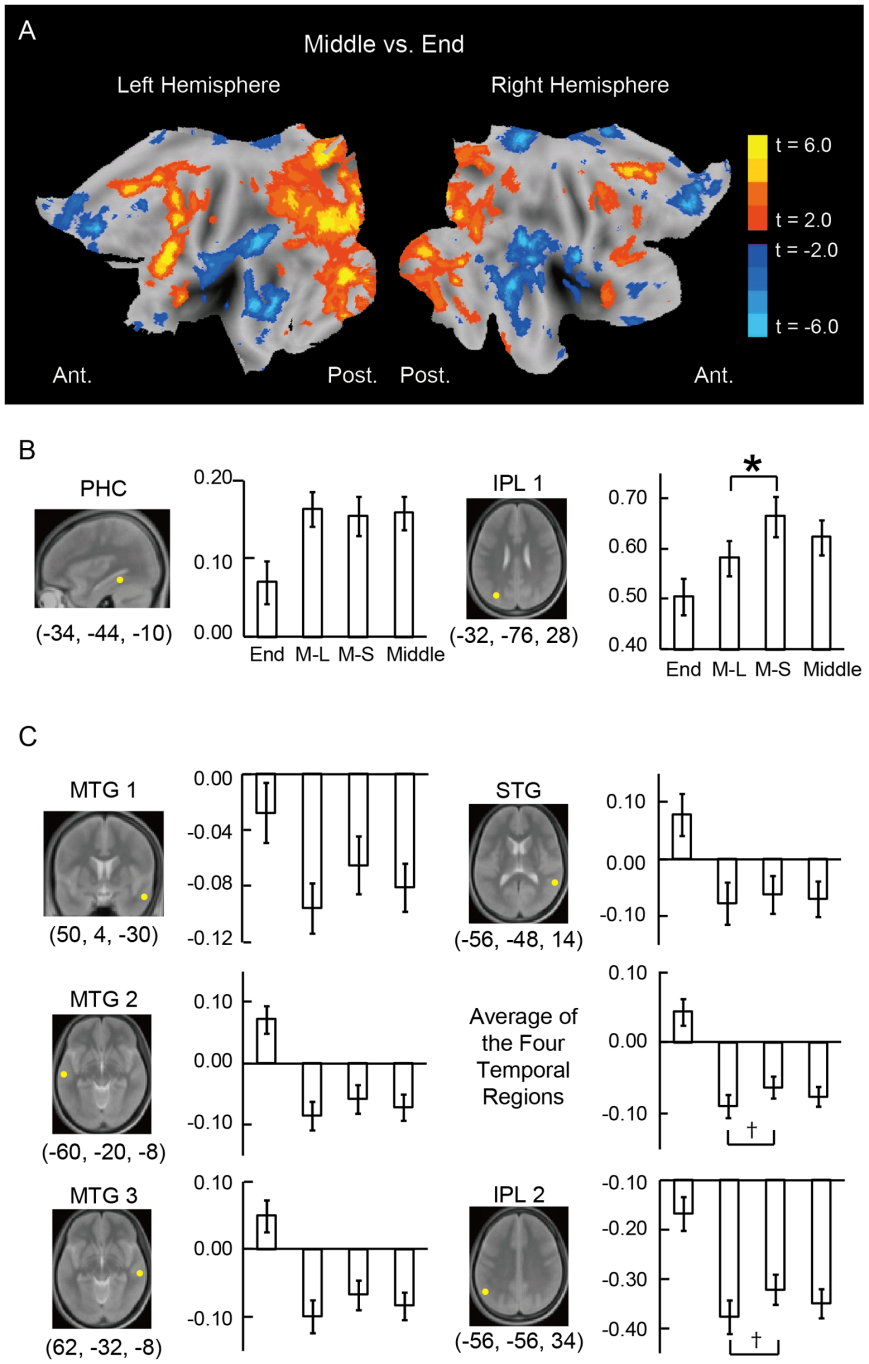
The brain activations associated with relational and item-based recency judgments. A) Statistical activation maps for signal increase in the contrasts Middle minus End trials and End minus Middle trials. Two-dimensional activation maps were generated using Caret [92]. Statistical significance is indicated using the color scale to the right. B) The magnitude of the brain activity during End, M-L, M-S and Middle trials relative to fixation in PHC and IPL1 that were activated during Middle vs. End trials. *: P<.05. C) The magnitude of the brain activity during End, M-L, M-S and Middle trials relative to fixation in the lateral temporal (MTG1, MTG2, MTG3 and STG) and IPL2 that were activated during End vs. Middle trials. †: P  = .08.

Whole-brain exploratory search did not reveal any significant signal change between M-L and M-S. Therefore, M-L and M-S trials were averaged into Middle trials in subsequent whole-brain analyses. As shown in Fig. 3A, the contrast Middle minus End, which is expected to reflect relational recency judgments, revealed significant signal increase in several regions including the left parahippocampal (PHC) and the left lateral parietal (IPL1 and IPL3) regions. The contrast End minus Middle, which is expected to reflect item-based recency judgments, revealed significant signal increase in several regions including the right anterior lateral temporal (MTG1), the posterior lateral temporal (MTG2, MTG3 and STG) and the left lateral parietal (IPL2 and IPL4) regions. Full significant brain activations are listed in Table 1.

**Table 1:** Brain regions showing signal increase in the contrasts Middle minus End and End minus Middle.

	X	Y	Z	t	Area	Label
Middle minus End						
Temporal Cortex	−34	−44	−10	4.2*	PHC	PHC
Parietal Cortex	−28	−76	40	5.5	IPL	IPL3
	−18	−64	30	5.5	PCu	
	−32	−76	28	4.1*	IPL	IPL1
Others	−18	−82	−16	6.5	ESC	
	−46	18	28	6.2	IFG/MFG	
	−12	−64	18	6.1	CG	
	−28	−90	12	5.9	ESC	
	32	−50	−32	5.8	Cerebellum	
	−2	16	52	5.7	SFG	
	−34	0	56	5.3	MedFG	
End minus Middle						
Temporal Cortex	−60	−20	−8	7.6	MTG	MTG2
	62	−32	−8	6.3	MTG	MTG3
	56	−48	14	5.8	STG	STG
	50	4	−30	3.6*	MTG	MTG1
Parietal Cortex	−54	−46	56	6.7	IPL	IPL4
	58	−38	50	6.6	IPL	
	54	−52	32	6.5	IPL	
	−56	−56	34	5.8	IPL	IPL2
	6	−48	42	5.4	PCu	
Others	0	60	10	6.4	MedFG	
	56	−10	12	5.9	PreCG	
	4	38	−2	5.5	CG	

* : small volume correction. PHC: parahippocampal cortex, IPL: inferior parietal lobule, PCu: precuneus, ESC: extrastriate cortex, IFG: inferior frontal gyrus, MFG: middle frontal gyrus, CG: cingulate gyrus, SFG: superior frontal gyrus, MedFG: medial frontal gyrus, MTG: middle temporal gyrus, STG: superior temporal gyrus, PreCG: precentral gyrus.

As shown in Fig. 3B, in the regions activated during Middle minus End trials, the contrast M-S minus M-L did not reveal significant signal difference in the parahippocampal region (PHC), but revealed marginally significant signal increase in the left lateral parietal region (IPL1) (t(71)  = 2.6, P<0.05). On the other hand, in the regions activated during End minus Middle trials, as shown in Fig. 3C, the signal difference between M-S minus M-L was close to significance in the lateral temporal regions (MTG1, MTG2, MTG3, STG) only when the four regions were averaged (t(71)  = 1.8, P = 0.08), and in the left lateral parietal region (IPL2) (t(71)  = 1.9, P = 0.06).

**Figure 4:**
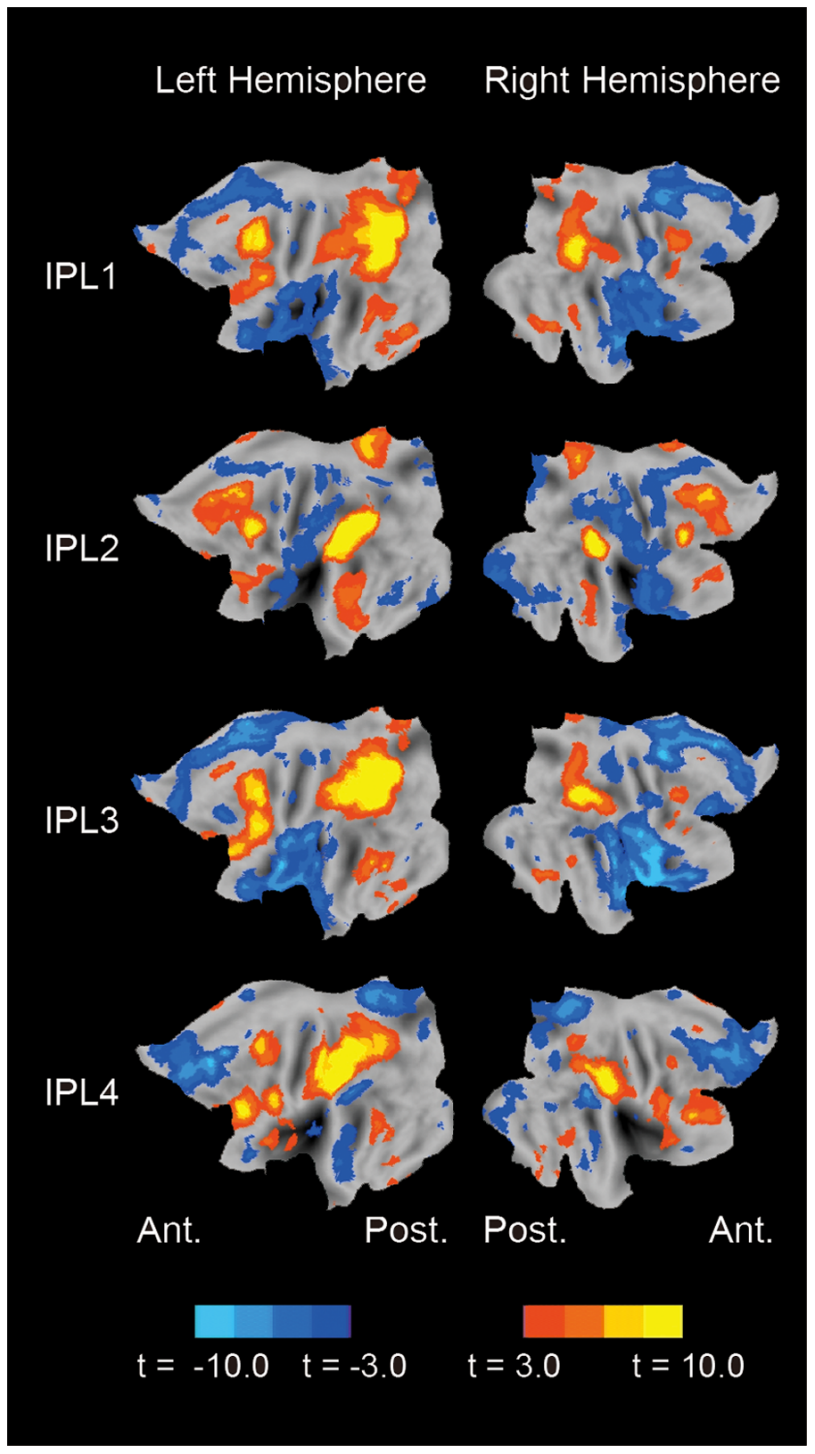
Whole-brain connectivity maps based on resting state data. The seeds were placed on the four activations (IPL1-4) during relational/item-based recency judgments. The format is similar to Fig. 3A.

To inspect the RSFC networks that the IPL regions belonged to, whole-brain connectivity maps were calculated based on the resting-state data set, with the IPL1-4 regions as seed ROIs (Fig. 4). The IPL1 and IPL3 belonged to the default mode network, and the IPL 2 belonged to the fronto-parietal control network and the IPL4 belonged to the dorsal attention network, based on Spreng et al [79].

**Figure 5:**
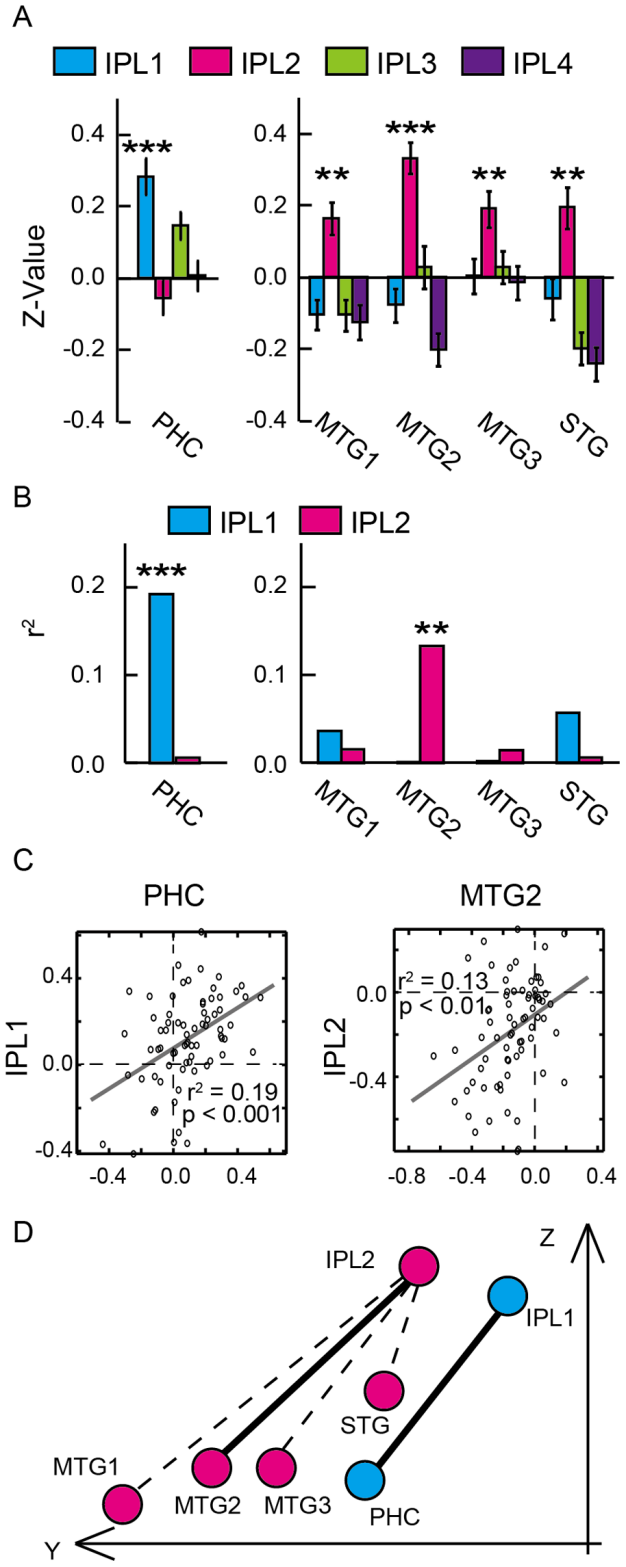
The RSFC and the task-related functional connectivity between the regions associated with recency judgments. A) The z-value of RSFC between five temporal regions (PHC, MTG1, MTG2, MTG3 and STG) and four left lateral parietal regions (IPL1, IPL2, IPL3 and IPL4). ***: P<.001, **: P<.01. B) Task-related functional connectivity of the brain activity during Middle minus End trials between five temporal regions (PHC, MTG1, MTG2, MTG3 and STG) and two left lateral parietal regions (IPL1 and IPL2) that showed significant RSFC. ***: P<.001, **: P<.01. C) Scatter plots of functional connectivity of the brain activity during Middle minus End trials. Correlations between PHC and IPL1 (left panel) and between MTG2 and IPL2 (right panel) are shown. One dot represents one subject. D) Two-dimensional (Y–Z space) spatial relationship of the temporo-parietal RSFC networks. Thick lines indicate significant task-related functional connectivity. Dashed lines indicate significant RSFC, without significant task-related functional connectivity.

We next examined RSFC between the brain regions in the temporal and parietal cortex. The ROIs were selected as follows: For relational recency judgments, the parahippocampal region (PHC) that was activated in the temporal cortex, and the two regions (IPL1 and IPL3) that were activated in the left lateral parietal cortex were selected. For item-based recency judgments, the four lateral temporal regions (MTG1, MTG2, MTG3 and STG) that were activated in the temporal cortex, and the two regions (IPL2 and IPL4) that were activated in the left lateral parietal cortex were selected. As shown in Fig. 5A, significant RSFC was found between PHC and IPL1 [t(50)  = 5.5, P<0.001, corrected for Bonferroni multiple comparisons of 5 temporal regions×4 parietal regions that were activated during relational/item-based recency judgments] and between the four lateral temporal regions (MTG1, MTG2, MTG3, STG) and IPL2 [smallest t(50)  = 3.8, P<0.01, corrected for multiple comparisons of 5×4]. The RSFC between PHC and IPL3 was significant only when multiple comparisons were not corrected [t(50)  = 3.1, P<0.005 uncorrected].

We then tested whether the significant RFSC-based networks between the temporal and parietal regions showed significant functional connectivity of the task-related activity. Across-subject correlation between the temporal and parietal regions was calculated based on the signal magnitude during Middle minus End trials. As shown in Fig. 5B, the task-related functional connectivity was significant between PHC and IPL1 [r^2^ = 0.19, t(70)  = 4.1, P<0.001, corrected for multiple comparisons of 5] and between MTG2 and IPL2 [r^2^ = 0.13, t(70)  = 3.3, P<0.01, corrected for multiple comparisons of 5]. Figures 5C is a scatter plot that demonstrates significant task-related functional connectivity between the temporal and parietal regions. Figure 5D summarizes the results by demonstrating that only the two combinations of the temporal and parietal regions showed significant task-related functional connectivity out of five temporo-parietal RSFC networks.

## Discussion

The present study examined whether the RSFC-based temporo-parietal memory networks showed functional connectivity of brain activity associated with recency judgments. The parahippocampal and lateral parietal regions in the RSFC network associated with relational recency judgments showed significant task-related functional connectivity. However, out of the four temporal- parietal RSFC networks associated with item-based recency judgments, only one of them showed significant task-related functional connectivity. These results suggest that a specific set of the RSFC temporo-parietal networks are recruited during recency judgments to support relational and item-based retrieval processes.

Based on the brain activity in Middle and End trials, five regions in the temporal cortex were detected in the present study, one associated with relational recency judgments and four associated with item-based recency judgments. The activation in the parahippocampal cortex is consistent with previous studies of recollection during item recognition [2]–[7], [18] and with those of relational recency judgments [20], [56], [62], [67]. On the other hand, significant signal increase during item-based recency judgments was found in the left posterior lateral temporal regions. Although the anterior lateral temporal activations during episodic memory, including item recognition [49], [80], [81] and recency judgments [20], [56], have frequently been highlighted, the posterior lateral temporal activation during episodic memory has rarely been reported. The posterior lateral temporal cortex has most often been implicated in category-specific semantic knowledge [82]–[85]. Although exact localizations of episodic and semantic memory in the posterior lateral temporal cortex may differ, the present study provides one example of the involvement of the posterior temporal cortex in episodic memory retrieval.

The left lateral parietal cortex has been implicated in retrieval success [5], [10]–[17], [86]–[88]. The present study revealed two left lateral parietal regions that showed significant functional connectivity with the temporal regions associated with relational and item-based recency judgments. The parietal region associated with relational recency judgments was located more ventrally to the parietal region associated with item-based recency judgments. This pattern of functional organization in the left lateral parietal cortex is consistent with that revealed in previous studies of item recognition where it has been demonstrated that the ventral part of the left lateral parietal cortex is associated with recollection, whereas the dorsal part is associated with familiarity [5], [11]–[17], [86]–[88]. Thus, although more investigations are needed to compare the exact functional organization of the left lateral parietal cortex, the present results support the view that relational and item-based recency judgments share their neural mechanisms with recollection and familiarity processes of item recognition. An alternative interpretation can be made based on the parietal attentional hypothesis [22]. Because the ventral parietal activation reflects memory recovery through interaction with the medial temporal lobe [22], the dorsal parietal activity was heightened during item-based recency judgments where the lateral temporal cortex was more recruited than the medial temporal lobe.

One caveat regarding the activations during Middle minus End and End minus Middle trials relates to the difficulty of task performance in these types of trials. The order of correct performance was End > M-L > M-S trials. However, the magnitude of brain activity in these trials (Fig. 3B and 3C) is largely inconsistent with the difficulty account: The magnitude of brain activity was significantly different between End and M-L trials, but the magnitude was almost the same or mildly different in the opposite direction between M-L and M-S trials. One exception to this pattern of brain activity is IPL1 (Fig. 3B), which showed the brain activity pattern consistent with the task difficulty. Although we could not completely exclude the difficulty account of this activation, the functional organization of the left lateral parietal cortex suggests the role for this ventral parietal region in recollection and relational processes.

The present study revealed two temporo-parietal RSFC networks that showed significant task-related functional connectivity between the temporal and parietal cortex. The network between the parahippocampal and the ventral parietal regions associated with relational recency judgments topographically corresponds to the medial temporal lobe subsystem of the default network [89] and to the default network [79], [90]. On the other hand, the network between the lateral temporal and the dorsal parietal regions associated with item-based recency judgments topographically corresponds to the dorsal medial prefrontal cortex subsystem of the default network [89] and to the frontoparietal control network that couples with both the default network and the dorsal attention network [79], [90]. The topographical correspondence suggests that relational recency judgments rely on the network that implements internal construction based on past episodes, whereas the item-based recency judgments rely on the network that implements attentional processes in decision making based on subtle difference of information available from the two words visually presented in the screen.

It has been demonstrated that activated regions that are detected by a contrast of test vs. control condition tend to be functionally connected to one another based on RSFC [36]–[44], [91]. The temporo-parietal RSFC results of the present study are consistent with those of the previous studies. The results of the present study also showed that the task-related functional connectivity is significant only in a subset of RSFC-based networks. Specifically, the significant task-related functional connectivity was observed between the left parahippocampal region and the left lateral parietal region and between the left posterior lateral temporal region and the left lateral parietal region. These results suggest that the right anterior temporal region that has been implicated in item-based recency judgments [20] is not essential for the function.
